# A Doctor’s Shop of the Olden Time

**Published:** 1887-11

**Authors:** 


### A DOCTOR'S SHOP OF THE OLDEN TIME.

The following sketch was made about fifty years ago by Dr. Daniel Drake. Early associations enable us to testify to its accuracy. The spirit of progress which has swept away so many of the customs and habits of the "good old times" before the war has probably left but few specimens of the old country doctor or of the shop, the theatre of his beneficent ministrations.

Our esteemed friend, Dr. E. W. Lane, of the Georgia Medical Association, has, doubtless, noted in his memory the progressive development of the shop into the elegant and well-appointed office of the present :

"My firm opinion is that one of the causes which retard us in raising our profession, in the West and South, to an excellence which at present seems almost hopeless, is the style in which our offices are fitted up and kept. Who can read and think with method, or sound logic, while everything around him is dirty and disordered ; his little stock of furniture displaced, as if a riot had just passed away ; his books scattered on chairs, tables and the greasy medicine shelves; in his bookcases volumes of different sets mixed together, some lying flat, and some, like the ideas of their reader, upside down; his skeleton exposed, and joint after joint torn off; his medicines unlabeled and thrown into a chaos as great as a treatise on the materia medica in the fourteenth century; bundles untied and bottles left uncorked, or stopped with plugs of paper; dead flies in the ointment within his jars, while others are wading through that which has so long spread over his counter that their feet are blistered by its rancidity; his spatulas foul and rusty; his scales tied with strings and balanced with pieces of paper; his mortar about as clean as the ancient Kentucky hominy-block, which, in the same day, contained the food of the family and of the family cow and horse, as it stood convenient to all the parties, on neutral ground, near the door of the cabin; his surgical instruments oxidated and rusting away like his mind; his study-table covered with loose papers and medical journals with their covers torn off; his walls over spread with a tapestry of cobwebs; his windows as opaque from the dust as the painted glass of an ancient cathedral; his foul candlestick standing all day on his lexicon, and his floor spotted over with the blood of his surgical patients and his own tobacco juice? The human intellect cannot act when thus encompassed. Ideas will not arrange themselves, nor will their foul surfaces cohere. The scene reacts upon his mind and a chaos within rivals that without."

### ITEMS.

GENERAL FRANCIS A. WALKER'S article on "What Shall We Tell the Working Classes," in the November *Scribner's*, will contain some very vigorous and plain-spoken words on the labor question.

DR. W. C. DEANE, New York, reports very favorable results in the treatment of sea-sickness from the use of large doses of bromo-soda. He himself found great relief from its use when other remedies had failed.

AT the latest advices yellow fever still prevails at Tampa, Florida. This epidemic appears to have been of mild character, there not having been more than two or three hundred cases in all, and has not spread beyond the limits of the city.

IT is said that there once existed "The Central College of Rochester." The faculty of this remarkable institution consisted of the students—the students of the faculty. At the close of a short and comprehensive course of study, the faculty conferred degrees upon themselves, and some are practitioners today.—*Ex.*

MEDICAL TEACHING AT OXFORD.—Oxford University has recently erected and equipped buildings devoted to the study of anatomy and physiology with histology. The future student of medicine can now ground himself thoroughly in these branches and in chemistry, at Oxford, before entering a college of medcine.—*Medical News.*

THE UNIVERSITY OF PENNSYLVANIA.—At the monthly meeting of the Board of Trustees of the University of Pennsylvania, on Monday, October 3d, Dr. Samuel G. Dixon was elected Professor of Hygiene in the Auxiliary Faculty of Medicine, in place of the late Dr. N. Archer Randolph, and Dr. A. H. P. Leuf was elected Assistant Director of Physical Education.

FOR SALE.—The following instruments, the property of the late Dr. James A. Gray: 1 Otis' urethrometer, 1 Thompson's divulsor, 1 Otis' urethrotome (curved), 1 Maisonneuve's urethrotome (curved), 1 Potain's aspirator, 1 American tonsilotome and various minor instruments. Some are new. All are in good condition. For full information address Dr. A. B. Ashworth, No. 30½ Marietta street, Atlanta, Ga.

MINISTERS GAINING COURAGE.—The Rev. Samuel P. Jones, in a recent lecture in this city on the subject, "Mothers, Homes and Heaven," expressed the pain he felt in visiting houses where he was surrounded by lap-dogs, Canary birds, parrots *and nothing else*, "and now you all know what I am talking about and what it means." Recent newspaper reports tell us that in a northwestern city eleven dead babies were found in one cistern.

DURING the Piedmont Exposition we had the pleasure of a visit from J. A. Reagan, A. M., M. D., of Weaverville, N. C., a member of the Board of Medical Examiners of that State. We are glad to learn from Dr. Reagan the successful workings of this board, and especially pleased to hear of the high stand which the graduates of the Atlanta Medical College have taken before it. Examining boards of like character and objects of those of North Carolina and Alabama are demonstrating their importance and usefulness, and, it is to be hoped, will soon be organized in every State of the Union.

A WEEKLY newspaper, published in this city, reports an operation of partial resection of the lower jaw as performed by a physician of this city. The statement is made that this is the most remarkable operation in surgery and one very rarely performed. Of course the object of such gratuitous and erroneous statements is to advertise the superior skill of the operator. Whilst the public may be entertained with such remarkable feats of surgical skill, the physician who appears as the hero of such exploits receives, we think, a doubtful benefit. We wonder why any respectable physician will allow himself to appear as the hero of such cheap notoriety. If the information was furnished by the operator to the reporter, he should have had some regard for truthsake. If the reporter concocted the story, he should repudiate such acts of hero-worship. Will not physicians rise superior to such clap-trap, and relegate this claim to newspaper notoriety to the quacks, who are compelled to employ such methods to sell their wares?—*Md. Medical Journal.*

AVULSION OF IN-GROWING NAIL UNDER HYPNOTISM.—Dr. S. L. Trivus, of St. Petersburg, relates (*Vratch*, No. 31, 1887, p.

607) a case in which he removed an in-growing nail from the great toe of a cook after a hypnotic state had been induced in the patient. The operation lasted about twenty minutes. At first the woman occasionally moved her foot about, but when the author had suggested to her that no more pain was to be inflicted, and that the foot must be kept at rest, she sat quite quiet till the matrix of the toe was incised. At that point of the operation, she shrieked out, and when questioned about the cause, stated that "a dog had just bitten her." After applying the dressing, Dr. Trivus woke her up and asked whether she would consent to the operation. She hesitated a little and then said, "Yes, go on." On his pointing to her bandaged foot, however, she at once guessed that all was over already, and burst into laughter, followed by hysterical sobs. No pain was felt till the next day, when she became somewhat lame. Dr. Trivus also relates an instructive case in which a young man of twenty-five, after having been hypnotized for the sake of experiment and subsequently awakened, could not return to his normal state for several hours; when left alone, he was seized with a kind of hypnosis, and on his way home fell into a deep swoon in the street.—*British Medical Journal.*

HYPODERMIC INJECTION OF CARBOLIC ACID.—Professor Benedikt has published a paper in the *Internationale Klinische Rundschau* on the Use of Subcutaneous Injections of Carbolic Acid. At the instance of Dr. Kunze, he had tried, about seventeen years ago, injections with a 2 per cent. solution of carbolic acid, in cases of rheumatism of the shoulder, with surprisingly good results. The joint, which was very painful on passive motion before the injection, was found, a few minutes after the injection, to be freely movable, as if it had been narcotized. The result was the more surprising, as it was known that subcutaneous injection of morphine had not so speedy an effect in such cases. In affections of the joints, in which there were great tenderness on pressure and distinct swelling of the bones, the morbid process disappeared in a few days after the injections. This was especially true of recent cases ; but even in old and complicated cases, the effect was not merely a passing one, but lasted sometime. Professor Benedikt also tried subcutaneous injections of carbolic acid in cases of polyarthritis, and in the first cases which he had observed the success was very striking. When the injection was made in the neighborhood of the affected joint, the pain not only quickly diminished, but the high temperature and the pains in the other joints also decreased. The course of the case was the same as after the administration of large doses of the salicylate of sodium. Both agents had a general and also a local influence, but the cause of the disease was only gradually eliminated from the body, and, in Professor Benedikt's opinion, this elimination was scarcely accelerated so much by the administration of the salicylate of sodium as by subcutaneous injections of carbolic acid. Professor Benedikt, therefore, advocated this plan of treatment in polyarthritis. The evil effects of the salicylate of sodium on the appetite, and in many cases also on the auditory apparatus and the whole organism, were well known. When both modes of treatment were combined, the salicylate of sodium being administered in small doses, and one to three subcutaneous injections of carbolic acid being given in twenty-four hours, the course of the affection was very much accelerated, and no bad consequences were observed, especially if the treatment was carried out from the very beginning of the disease. Extraordinarily good results were obtained by the method in cases of inflammation of the sheaths of tendons, especially after injury. A few injections sufficed to cut short the morbid process, and no local pain or muscular atrophy, etc., was observed, provided the disease was treated in the above-mentioned way from the very first outset. Subcutaneous injections of carbolic acid also gave Professor Benedikt good results in cases of pain due to club-foot, as well as in local periosteal inflammation, in spondylitis, and in scrivener's palsy. In most cases he had given only one injection a day, but in cases of polyarthritis he had given from three to five injections in the course of twenyt-four hours. The local anæsthesia which occurred in two cases (lasting in one of them for several months) was due to the effect which the fluid exerted directly on cutaneous nerve-trunks.—*British Medical Journal.*

NATIVE DOCTORS IN MADAGASCAR.—The English and Norwegian missionaries in Madagascar have organized a "Medical Missionary Academy" in order to secure to the medical students of the several missions a greater variety and thoroughness of instruction. Students are required to pass an entrance examination in the three "Rs," geography, and some foreign language. The medical curriculum is to extend in future over five years, and will afford opportunities for obtaining at least an elementary acquaintance with all the subjects of medical education. Great stress is wisely laid in the syllabus on the practical side, and in the examinations also prominence is given to the oral and practical parts. There will be two examinations: the first at the end of the second year of the curriculum, in anatomy, physiology pharmacology and therapeutics; the second, comprising the remaining subjects of the curriculum, at the end of the fifth year; the successful candidates will receive the title of "Member of the Medical Missionary Academy." The new regulations will be gradually enforced with a gentle hand, the examiners being required always to bear in mind that the great object of the examination is to test the fitness of the candidate to undertake the ordinary work of a Malagasy doctor.—*British Medical Journal.*

DR. J. G. FAIRCLOTH, of Faircloth, Ga., was married, October 16th, 1887, to Miss Ida C. Stubbs, a daughter of Rev. H. Stubbs, of Branchville, Ga.

ON the medical books of the Driffield Union are the names of twenty persons whose united ages amount to 1,530 years, or an average of 76½ years each patient. Amongst them are nine septuagenarians, seven octogenarians and four nonagenarians, the oldest being 97.—*Medical Press.*

DR. WOODBURY has associated with him Dr. Waugh, and the two have assumed the joint editorship and properietorship of the *Philadelphia Medical Times.* Of their ability to edit the Journal there is no doubt, and we trust that the proverbially poor business qualifications of doctors may find in them a bright and shining exception.—*Ex.*

"EXCUSE me," said the doctor, as he halted a citizen on the street, "but I have a sure and speedy cure for that mild form of erysipelas in your face. It is only $1 a bottle." "Erysipelas!" howled the other. "I'll warrant a cure in a very few weeks." "You old idiot, don't you know nothing?" shouted the enraged man. "Erysipelas! why, I brought this face on with whisky. Am I to be insulted after working as I have for the last twenty years? Go on, sir, or I'll do you serious injury."—*Ex.*

A DRUG CLERK PUNISHED.—James A. Stewart, of Wichita, Kan., was sentenced on September 22, to seventeen years and four months imprisonment in the county jail and fined $20,800, with costs of prosecution, for the violation of the prohibition law. Stewart was a clerk in the West End drug store, and plead guilty to an indictment containing 2,080 counts. The punishment imposed on Stewart is the heaviest ever given in the State for violation of the liquor laws.—*Exchange.*

THE AMERICAN CONSTITUTION.—The best reason for American pride in the Constitution lies, not in the creative genius of its framers, nor in the beauty and symmetry of their work, but in the fact that it was and is a perfect expression of the institutional methods of its people. It is for that reason that it meets their needs as well to-day as in 1787-89. So long as they shall continue in the ways of their fathers; so long as they shall regard with pronounced disfavor the political quacks who constantly beg them to hazard a trial of never-tested remedies, so long may they continue to take a just pride in their Constitution, under all its possible coming changes, as one which has been "adequately discussed," and the results of discussion have been fully "tested by experiment."—*Alexander Johnston in New Princeton Review for September.*

THE ROYAL WESTMINSTER OPHTHALMIC HOSPITAL.—On Friday, October 7th, the winter session was opened by the first of a monthly series of clinical evenings. There was a very large attendance of cases, among which we may mention the following: Detached retina, retinitis pigmentosa, retinitis albuminurica, retinal hæmorrhage, optic neuritis, choroiditis from inherited syphilis, progressive myopia with posterior staphyloma, persistent pupillary membrane, and congenital malformations of the optic disc. Ophthalmoscopic lesions are so seldom met with in general practice that, however *au fait* with the ophthalmoscope a practitioner may have been in his student days, his diagnostic powers must become a little rusty in course of time, unless he takes an occasional opportunity of seeing typical examples of the more common affections. A collection of cases, such as that on Friday, affords just the opportunity that is wanted, and we are sure that many practitioners will gladly avail themselves of these clinical evenings to renew their acquaintance with the ophthalmoscope.—*British Medical Journal.*

CREMATION IN PARIS.—The cremation apparatus in the cemetery of Père la Chaise, Paris, will, it is expected, be in working order in November next. This building, which is situated in the north of the cemetery, near the Jewish and Mussulman enclosures, has the shape of a parallelogram and is three stories high, surmounted by two chimneys in white stone. Over the facade are three domes. The facade will be in black and white marble, and a large vestibule is also to be erected. Dr. Brouardel, in a report which he has drawn up on the subject, estimates that the furnaces will be able to consume 4,500 bodies annually, which is said to be about the average number of corpses leaving the hospitals in Paris during the year. The Chamber of Deputies has already passed the cremation bill and it only remains for the Senate to do the same. Whether Parisians, who are outwardly so strict in the treatment of the dead, will be inclined to consign their deceased relatives and friends to the new crematory remains to be seen.—*British Medical Journal.*

COCAINE SOLUTIONS.—The results of my experiments may be summed up as follows:

1. Solutions of cocaine, even though admixed with boracic acid, gradually lose their anæsthetic power.

2. Freshly-made solutions, applied immediately, will never fail to give the most satisfactory results.

3. When freshly prepared, much weaker solutions will be needed than when kept on hand. A 2 per cent. strength is as effectual (when fresh) as a 4 per cent. two or three weeks old.

4. Fresh solutions are not productive of destructive inflammation to the eye, as has been attributed by some to older solutions.

It would probably be well to state that in these experiments its use has been chiefly confined to the male urethra. I always increase the strength of the solution in proportion to the pain to be produced. If a sensitive stricture is to be cut, I use a strength of from 10 to 20 per cent.; if an examination is to be made or a bougie introduced, a 2 to 4 per cent. answers every purpose.—*W. F. Glenn, M. D., in Jour Am. Med. Assoc.*

A NEW TREATMENT OF STERILITY.—In an article upon "The Obstacles to Fecundity in the Human Species" (*Journal de Médècine*, May 16, 1886), Professor Pajot says: Has the woman an anteversion? Say to her: "Have the kindness, if you please, every evening when you expect to have intercourse with your husband, not to urinate for five or six hours. Don't ask why; that doesn't concern you. Only don't urinate. You wish to have children? Yes? Well, then, urinate after intercourse, and not before." If she has a retroversion, say to her: "Madame, when your menses are over, eat plenty of eggs and plenty of rice. Take every night for three or four days a little pill which I am going to give you." (This little pill contains simply a third of a grain of extract of opium.) "Manage not to go to stool for three or four days. Then have intercourse with your husband, but don't go to stool till afterwards." You will say that all this is very ridiculous; yet the whole process is entirely rational and is based upon anatomical and physiological principles.

THE following is a copy of a beautifully engraved invitation card being received by favored ones in Barnesville, Macon and many other places where the parties are so well and popularly known in *elite* circles: "Mr. and Mrs. R. J. Powell invite you to be present at the marriage of their daughter Maybelle, to Dr. Robert O. Cotter, Wednesday evening, November second, eight o'clock, at their residence, Bonnie Castle, Barnesville, Georgia, 1887." And thus the same sweet, old story of love and happiness is again told, and rainbows of promise bend over the brow of the future. As the two pass down the aisles of time hand in hand and heart to heart, may fond effection ever wreathe sweetest and fairest garlands to adorn and span their pathway. The bride-expectant is the lovely and irresistibly-charming daughter of Col. R. J. Powell, the distinguished Senator of this district, able and popular Treasurer of the State Agricultural Society, the successful and very wealthy financier, and the esteemed citizen and Christian gentleman. She holds the palm for beauty over many fair contestants, and at her shrine the flower of knightly manhood delights to bow in love and admiration. She is one of those adorably lovely women of whom poets love to sing. The happy and exultant groom to be is a popular citizen and distinguished oculist of Macon, a young gentleman of superior polish and culture, remarkable mental attainments, and possessing a daily increasing practice in which he is successful. So fair a bride is indeed worthy of so excellent a groom.—*Macon News.*

HONORARY DEGREES AT WASHINGTON.—The hopes entertained by many visitors to the International Congress, at Washington, of obtaining honorary degrees do not seem to have been fulfilled in any sense of the term. There is a general impression in Europe that such degrees are to be obtained without too much difficulty, and although this may be the case to a great extent, it would appear that no provision has been made in the charters of the various collegiate bodies whereby the authorities are empowered to confer honorary degrees, and consequently no such distinctions were distributed. It is said that this distressing inability to comply with a very general custom elsewhere was due to the fact that the men in whose power it was to do the needful were conspicuous by their absence from the Congress, and there may be some truth in this assertion. In any case the only thing of the kind which has come to our knowledge was that of an English practitioner who contrived to get himself examined at Washington. The Medico-Chirurgical College, at

Philadelphia, is reported to have elected a certain number of honorary fellows, but no publicity has been given to the elections, and the choice has therefore only a restricted and personal interest. Somewhat more difficult to explain was the lack of initiative on the part of the medical societies in the matter of electing honorary fellows. This is an easy compliment to pay, and is generally practiced on a liberal scale in Europe. The reticence of the American medical societies in this respect savors either of impotence or disrespect.—*Medical Press and Circular.*

FEVER IN LONDON.—It may be interesting to note here the progress of the present epidemic of scarlet fever in London. During the second quarter of the present year there were 606 patients admitted to the hospitals of the Metropolitan Asylum Board; during the month of July there were 349 further admissions; during the fortnight ending August 13th, 217; and during the weeks ended August 20th, upward of 100; August 27th, 158; September 3rd, 173; September 10th, 200; September 17th, 203; September 24th, 277; October 1st, 318. On September 25th there were 12 admissions; on September 26th, 69; September 27th, 58; September 28th, 47; September 29th, 30; September 30th, 45; October 1st, 40; October 3rd, 53; October 4th, 62; October 5th, 46. During the week ended October 1st, the daily excess of admissions over discharges and deaths were 32; hence the hospitals have become rapidly filled. Of these admissions about 60 per cent. came in under the poor-law officials, 29 per cent. under the sanitary authorities, 8½ per cent. from the general hospitals, and 2 per cent. from private medical practitioners. In this connection it is worth pointing out, as did Sir E. Galsworthy at the recent meeting of the managers, that the cost of preparing accomodations for the two classes—pauper and non-pauper—of afflicted persons virtually comes out of the same pocket, the ratepayers paying alike to the vestries and the guardians. On Wednesday there were 1,812 patients in the hospitals, including 1,673 scarlet fever, 105 enteric fever, 7 typhus, and 27 other diseases. In accordance with the resolution passed at the special meeting of the board on Monday last, huts are being rapidly erected on the grounds belonging to the Southwestern and Northwestern Hospitals.—*British Medical Journal, October 8th, 1887.*

PROFESSIONAL ETIQUETTE.—The following rules have been adopted by the medical circle of Verviers:

1. A physician called to attend the patient of another shall not render his services until the colleague who preceded him shall have been discharged, and in case he has not been discharged shall propose a consultation.

2. The physician called in an emergency to a patient under treatment ought to limit himself to giving necessary attention, and leave a copy of his prescription for the attending physician.

3. All the members of the circle agree to take the place of an associate who is taking a vacation or detained at home by sickness, but this is not to hinder him from making an agreement with some of them for attention to his *clientele.*

4. The physician so absent or sick ought to advise his associates of the date at which he will resume his visits; the latter shall immediately transmit to him the list of patients treated by them, with such information as they may think indispensable.

5. It is required by the dignity of the profession that the patients of an absent or sick associate should not be retained.

6. Another duty of the physician is that he shall abstain from unfavorable comment on the treatment of the brother physician who has preceded him and from unkindly insinuations.

7. A physician who succeeds another is not permitted to adopt for his *client* a scale of fees below that of his predecessor.

8. The consulting physician shall not supersede the attending physician until after having first apprized him in writing of the intention formally expressed by the patient.

9. The physician ought to leave to the patient the choice of the consultant and of the *accoucheur*, and to depart from this line of conduct only when the family earnestly requests him to designate for himself.

10. The physician expelled from the circle is the only one with whom a consultation may be refused.—*American Practitioner.*

CALCIFICATION OF A PORTION OF THE PENILE URETHRA.—Some months ago I was consulted by a man, aged 60, who had all the symptoms, both subjective and objective, of stone, and, as he refused to allow either lithotrity or lithotomy, and insisted on my systematically administering various lithontriptics, with the hope of ultimately dispelling by this means the cause of his trouble, notwithstanding my assurance to the contrary, I consequently had an opportunity of observing the following noticeable features of his case, which did not develop until he was over two months under treatment.

During one of his visits, he drew my attention to a small nodule on the under surface of the penis, which on examination I found to be a hard, gritty substance, measuring nearly a quarter of an inch in its long axis, and apparently filling up the urethra almost completely, as the smallest sized catheter could not be insinuated beyond the point of induration. The patient states that since he noticed this kernel (as he termed it), the pain has become more constant, and is always referred to the neck of the bladder; the stream of water is not interrupted suddenly as before, but is much narrower, and passes with greater difficulty and pain. The last time I saw him it had increased to the extent of half an inch, and felt harder, the anterior portion being thinner than the posterior, and seemed to have assumed a spoutlike profile, the sides of which were adapted to the floor and lateral portions of the urethra.

The foregoing is, I believe, judging from the symptoms and course of the disease, a case of either (1) a hypertrophied horn of a mulberry calculus, which projects into the urethra, but remains attached to its nucleus in the bladder; or else (2) a center of calcification originated *per se* in the urethra itself, probably from a detached portion of calculus getting lodged in a neighboring lacuna on its journey through the vesical outlet. It is unfortunate in the interest of pathology that the man would not submit to active treatment, when all doubts could have been set at rest. I have lately lost all traces of him.—*British Medical Journal.*

DR. JAMES A. GRAY IS DEAD.—This startling announcement, a few days ago, was a shock, and carried sorrow to many hearts. Not only in his native State and in the city of his adoption was it felt, but by many of the profession throughout the land who knew him well and loved him. In Atlanta he was almost idolized by all classes of people. Especially was he held in high esteem and affectionate regard by his professional brethren ; they recognized in him their peer in every relation of life, and loved him for his brilliant, almost transcendent attainments as a medical man, no less than for his beautiful life and character—that of a Christian gentleman. "Pure as a pearl and as perfect," if ever truthfully written of any man, could be appropriately inscribed on the spotless tablet which shall commemorate his life—a life gentle and full of Christian charity—replete with deeds worthy of record above. That "death loves a shining mark" was never more fully and fearfully verified than when it struck down this cherished, beloved man, so young, so strong and so good—a man endeared to all who knew him. He was the friend of the poor, the solace of the afflicted, the counsel and companion of his colleagues, the prop and stay, the pride and hope of his aged parents, and, alas! the only one on earth (but, thank God, one remains—their faith and hope, that in the bright Beyond they shall meet their three "boys," where partings are not known).

Atlanta put on her weeds of deepest woe that day, and the community, as one man, mourned the loss of their favorite citizen. There was the twang of an invisible bow; a silent arrow sped, and a giant-heart was stilled forever! Death is at all times terrible; but when, with its invisible hand, it strikes down by our side our brightest and best, defying physical laws and mocking at manhood, respecting neither the helpless loved ones nor vigorous youth, nor the tender nor the aged, we tremble, as well we may, and with heads bowed with reverential awe, exclaim, "thy will be done, oh God, not mine!"

Peace to his ashes. "Green grow the turf o'er thy grave," brave, noble, generous Gray! A stranger—one who never was blessed with even seeing him, yet who, by correspondence, had become acquainted with his nature, and had learned to love him as a brother—a confrere away out here in the wilds of Texas (as the Georgia people would say), mingles his tears and regrets with those who were blessed with his friendship, cheered by his companionship, and bereaved and *impoverished* by his most untimely death! He feels honored in the privilege of doing so.

Dr. Gray was only 37 years of age, and had practiced only ten years! Yet within that brief space he had crowded the work of a lifetime. Graduating at the Atlanta Medical College in 1879, he was, within *one year*, invited to a chair in its faculty; at one step he went to the front, and became a *leader* in the profession, whom his elders even delighted to recognize and to honor! As managing editor of THE ATLANTA MEDICAL AND SURGICAL JOURNAL, he had made a name for himself, which will endure while medicine is a science, and men respect those who lend it lustre, and give to its name both dignity and honor.—*Daniel's Texas Medical Journal.*

CURIOSITIES OF HYPNOTISM.—At a recent meeting of the Paris Academie de Medicine, M. Luys read a lengthy communication on hypnotism in reference to very curious experiments he made on different subjects. He found that he could produce at will on patients hypnotized intense and varied emotions of joy, sadness, terror, etc., without the subject having any consciousness of his acts after being awakened, and that by substances held *at a distance.* A tube filled with a solution of strychnine, applied to the back of the neck, left side, produced bilateral contractions, convulsive movements and contractions of the face. The same tube placed on the right side produced exactly opposite phenomena that is to say, all contractions disappeared and an expression of gaiety took its place. Spirituous liquors produced the same symptoms of inebriety as if they had been taken to a considerable extent. By taking away the tube all these phenomena immediately ceased to show themselves. Other substances, such as coffee, Indian hemp, beer, champagne, had the effect of making the patient exceedingly loquacious. His volubility was surprising, yet in spite of the lucidity of his answers, he was quite unconscious of his acts. Valerian plunged him into profound grief. He scrapes up the clay, kneeling, and the idea strikes him that he is in a cemetery after the exhumation of some dear departed friend. He pushes aside the sand with his hands, collects with reverence the bones into a small heap, upon which he places a crucifix, accompanying the little ceremony by groans, genuflexions, signs of the cross, etc. The Indian hemp produced great gaiety. A young girl, who was very fond of theaters, improvised a scene and sang pieces from an opera perfectly. Others again exhibit tendency to robbery, evasion, assassination, and describe in the minutest details the circumstances of the crime. M. Luys, in concluding his remarks on his highly interesting experiments, said that he could not conceal from himself the grave consequences that these new studies in experimental psychology may have for acts in social life. It is not only a question of extraordinary suggestions put to certain subjects, but a new order of medico-legal questions which, *á propos* of toxic medical substances, force themselves on the attention of the profession. This communication of Dr. Luys excited considerable attention in the assembly, and Dr. Brouardel insisted on the necessity of naming a committee to examine into the cases cited by M. Luys. It was not a question of individuals, more or less capable of being hypnotized, but of persons who could be poisoned by a substance which did not enter the body and lost none of its quantity. He thought that considerable danger might be incurred, as any one might be accused of killing a person without being able to prove his innocence. The Academie decided that a committee should be chosen at the next *séance*.—*Medical Press and Circular*.

CLEVER DETECTION OF A CRIMINAL BY A DOCTOR.—A rather novel point in forensic medicine has arisen in connection with the murder at the Rue Montaigne, Paris, the victims of which were a celebrated *demimondaine*, who, along with her maid and maid-servant, was found murdered in their apartments under circumstances of great cruelty. Their throats had been cut after a severe struggle, and their bedding and the walls of the apartments in their vicinity were copiously bespattered with blood. The unfortunate chief victim was the possessor of jewelry and other valuables to the extent of some thousand pounds, presumably the motive of the murder. These, however, were in an iron safe, and as the murderer, who was evidently single-handed, could neither open this nor carry it away, he had to leave without the wages of his crime. The key was subsequently found concealed in a wardrobe. A man named Pranzini, a follower of the victims, was arrested upon suspicion, but the closest examination of his clothes, both those which he had worn on the evening of the crime and those found at his residence, showed no trace of blood. This was relied upon as a strong point in the defence, but Dr. Brouardel, the *Médicinlégiste* engaged in the matter, recalled the fact that in the next apartment was found a toilet-stand upon which was a large basinful of ensanguined water, as if some person stained with blood had washed therein. As none of the three victims had presented the appearance of having done this, Dr. Brouardel conceived the idea that the murderer had, on entering the apartments, undressed himself, had committed the crime in a state of nudity, had then carefully washed himself of its traces, and had finally, all being completed, resumed his clothes, of course free from all sanguineous stains. Dr. Brouardel now demanded from the *juge d'instruction* an authorization to examine Pranzini totally undressed, and on this being accomplished found a long tearing scratch (*estafilade*) extending down the front of his right thigh. Interrogated upon this, the prisoner said that he had been attacked by a severe itching in the part and had torn himself in relieving it. Being invited to repeat the gesture, he did so, scratching himself from the knee towards the body, when the medical expert showed that the tear had been produced by nails, or at all events some sharp body, acting from above downwards in the direction of the knee, as was shown by the pointed fragment of epidermis projecting upwards from the lower end of the wound. The expert's hypothesis is that when the assailant approached one of his victims, with the weapon in his right hand and with his right side next her, she inflicted the injury with her nails. Questions of blood-stains on clothing have frequently formed important evidence in murder trials; but the present is probably the first instance of an ingenious plan having been adopted to escape clothing stains, and of its being defeated by an accident developed by the acuteness of the medical expert. Pranzini was convicted, without extenuating circumstances, and was sentenced to death by the guillotine.—*Dublin Journal of Medical Science.*—*Medical and Surgical Reporter.*

